# Fusion of Spectroscopy and Cobalt Electrochemistry Data for Estimating Phosphate Concentration in Hydroponic Solution

**DOI:** 10.3390/s19112596

**Published:** 2019-06-07

**Authors:** Dae-Hyun Jung, Hak-Jin Kim, Hyoung Seok Kim, Jaeyoung Choi, Jeong Do Kim, Soo Hyun Park

**Affiliations:** 1 Smart Farm Research Center, Korea Institute of Science and Technology (KIST), Gangneung-si 25451, Gangwon-do, Korea; jeoguss@gmail.com (D.-H.J.); hkim58@kist.re.kr (H.S.K.); jaeyoung.choi@kist.re.kr (J.C.); kimjeongdo@kist.re.kr (J.D.K.); 2 Department of Biosystems and Biomaterial Engineering, College of Agriculture and Life Sciences, Seoul National University, Seoul 08826, Korea

**Keywords:** phosphate sensing, Multi-sensor data fusion, hybrid sensor system, Feed-forward back-propagation ANN

## Abstract

Phosphate is a key element affecting plant growth. Therefore, the accurate determination of phosphate concentration in hydroponic nutrient solutions is essential for providing a balanced set of nutrients to plants within a suitable range. This study aimed to develop a data fusion approach for determining phosphate concentrations in a paprika nutrient solution. As a conventional multivariate analysis approach using spectral data, partial least squares regression (PLSR) and principal components regression (PCR) models were developed using 56 samples for calibration and 24 samples for evaluation. The R^2^ values of estimation models using PCR and PLSR ranged from 0.44 to 0.64. Furthermore, an estimation model using raw electromotive force (EMF) data from cobalt electrodes gave R^2^ values of 0.58–0.71. To improve the model performance, a data fusion method was developed to estimate phosphate concentration using near infrared (NIR) spectral and cobalt electrochemical data. Raw EMF data from cobalt electrodes and principle component values from the spectral data were combined. Results of calibration and evaluation tests using an artificial neural network estimation model showed that R^2^ = 0.90 and 0.89 and root mean square error (RMSE) = 96.70 and 119.50 mg/L, respectively. These values are sufficiently high for application to measuring phosphate concentration in hydroponic solutions.

## 1. Introduction

Hydroponic cultivation is a method for growing plants in which nutrients can be efficiently supplied to crops in the form of mineral nutrient solutions. This type of agriculture produces waste water containing large amounts of point source pollutants that are highly enriched in nitrate (400–1500 mg/L) and phosphorus (60–500 mg/L) [1,2]. To reduce the economic cost and minimize soil pollution in cultivation fields, there is an increasing need to recirculate and reuse nutrient solutions in a closed cultivation system. Traditionally, the nutritional management of hydroponic solutions used for greenhouse cultivation is conducted on the basis of monitoring a solution’s pH and electrical conductivity (EC) [3,4,5]. Using this method, the quality of a hydroponic solution is maintained through the automatic supply of nutrient solutions into the hydroponic reservoir in proportion to the declining EC of the solution resulting from the nutrient uptake of plants. However, one of the weakest aspects of the EC-based nutrient management system is the lack of information for managing individual nutrients in the solution. Thus, EC measurements alone do not provide sufficient information to manage optimal plant production from a solution fertility perspective [6].

### 1.1. Ion Monitoring in Hydroponic Solution

Many researchers have been actively studying the use of ion sensors to detect changes in the components of nutrient solutions. Bailey et al. [7] confirmed that electrode-type ion sensors can be used in a circulating hydroponic system. Gutierrez et al. [8] proposed an artificial neural network (ANN) model with the goal of using ion-sensitive field-effect transistor (ISFET)-type chemical sensors to detect major ion concentrations in a nutrient solution. Gieling et al. [9] also attempted to employ ISFET to observe ion changes in a nutrient solution. Bamsey et al. [6] proposed an ion sensor system that can be used in hydroponics via the optode ion detection method. These various types of ion sensor platforms have been used to detect ion components in nutrient solutions but they involve some problems regarding the durability and stability of ion sensors when replacing EC-based control methods [9,10,11]. Most ion sensors are difficult to use in locations with extreme changes in the external environment, such as greenhouses. Among these sensors, the most efficient sensor type is the ion-selective electrode (ISE) type and there have been cases in which a two-point normalization method was employed to successfully observe NO_3_, K and Ca ion components during a lettuce cultivation period [12,13,14].

### 1.2. Phosphate Detection in Hydroponic Solution

The absorption of phosphate ions is very important to crop growth and development, especially for fertilization and flowering. However, unlike other ion components, there are some limitations to phosphate measurement in hydroponic solutions. The hydrogen bond structures of phosphate ions change according to pH levels and precipitation problems occur with heavy metals such as Ca and Al. Therefore, it is very difficult to detect phosphorus in hydroponic solutions [15]. As such, sensor systems that can effectively detect phosphate ions in nutrient solutions have not yet been established. In addition, there has been insufficient development of ionophores, which allow for selective reactions with phosphate ions and can easily be manufactured. Therefore, the use of the electrode-type detection method is difficult for phosphate measurement. In the most similar approach, a solid cobalt surface is polished and soil samples are extracted using electrodes. This creates a reaction with phosphate ions to quantitatively detect the phosphate ions, for which a sufficient selective sensitivity could be confirmed [5,16,17]. However, the cobalt electrode has not yet been used in practical applications because of some limitations. To induce oxidation reaction on the surface of the cobalt electrode, frequent surface abrasion must be applied under constant conditions, which affects changes in the electrode signal band [5]. To resolve this problem, a technique has been proposed to adapt to the developed calibration equation [12]. Previous studies have shown that the simplified calibration line form has some limitations because the factors affecting the electromotive force generated by the phosphate ion concentration of the cobalt electrode are very complex in the mixed solution of various ions [18,19].

A method of optical analysis for selective detection at complex ionic concentrations has also been proposed [20,21]. Of the existing phosphate ion measurement methods, Visible, near-infrared diffuse reflectance (VNIR) spectroscopy has been used to successfully estimate several chemical components of solutions [22,23]. The most commonly used multivariate methods for chemical analysis are partial least squares (PLS) regression and principal component regression (PCR), where factors that relate to variation in the response measurements are regressed against the properties of interest [24]. Mouazen et al. [22] used a VIS-NIR band spectroscopy technique, which effectively detects soil ion components (carbon and phosphorous). In addition, Mouazen et al. [25] compared each prediction model developed via the principal partial least squares (PLS) method and their accuracies were compared. It was also reported that mid-infrared spectroscopy allows the detection of quantities of EC, pH, NO_3_, K and Ca ions in nutrient solutions [26]. Chang et al. [27] predicted total N and Ca ions in the soil using NIR spectroscopy with a PCR calibration model. However, several studies indicated that the performance of NIR spectrometry in quantifying phosphate concentrations in soil or solutions is generally poor [28].

### 1.3. Data Fusion Approach for Multi-Sensor System

**Figure 1:**
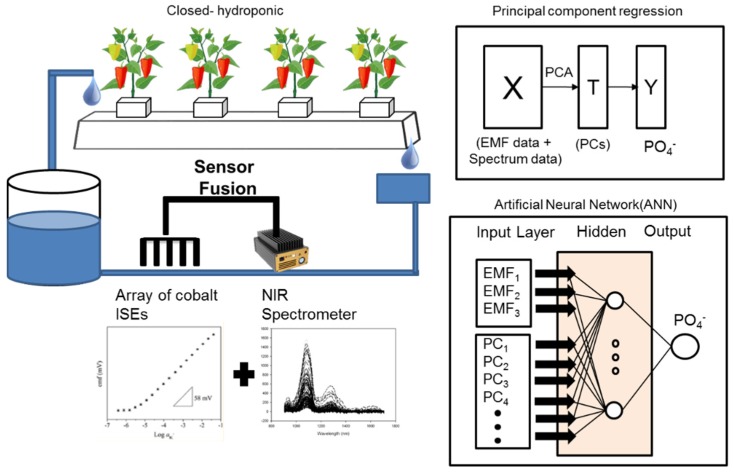
Concept art of newly developed calibration model combining near infrared (NIR) and cobalt electrodes using a multilayer neural network structure to detect phosphate concentrations in paprika hydroponic solutions.

The multi-sensor data fusion approach has been significantly highlighted in various fields because it can enhance not only the accuracy of sensors but also the robustness of a system [29,30,31,32]. These advantages have prompted researchers to explore new possibilities for acquiring sophisticated information to achieve new interpretations of various measurement data [33] (Figure 1). Therefore, the data fusion method has been applied to effectively improve the performance of the measurement system in various fields [34]. One of the areas that actively use data fusion is precision agriculture [23,35] and data fusion has been applied using various analytical sensors to establish soil mapping and analyze the nutrient status of soil [23,36,37].

In order to measure phosphate ions in the soil, La et al. [38] used data fusion methods applicable to nutrients rather than soil samples. Furthermore, a phosphate detection model had been developed using the multiple regression method and PLS regression by adding characteristic data of soil and much better results could be obtained through fusion. Nevertheless, the present study employed a linearized compensation method (e.g., two-point normalization, baseline correction) and developed multi regression models using the predicted results. The concentration values in the ISE and data obtained from various sensors were re-analyzed through multiple regression analysis. Therefore, the possibility of compensation by these processes could not be excluded. It is necessary to confirm the possibility of developing a model through the fusion of raw data from sensors and perform additional experiments to determine the possibility of applying this to hydroponic nutrient solution samples.

Currently, the most widely employed neural network structure is the multilayer perceptron structure and the learning performance has been significantly improved through the error back propagation algorithm [39]. Neural networks exhibit particularly good performance in highly nonlinear models [40,41] and it is possible to omit the step of designing a hypothesis for the model. Therefore, research on using ANNs in a variety of fields are now being actively pursued [42,43,44,45]. Yi et al. [46] found that the ANN method is generally more accurate than the linear regression method for estimating leaf N concentration when the same input variables are used. The relationship between ion concentrations of nutrient solutions and responses of the spectroscopy and electrochemical sensors is non-linear because it is very difficult to develop a linear calibration model reflecting interference by other ions in hydroponic nutrient solutions. As a successful example, Gutierrez [8] proposed the ISE based ion regression model using ANN and reported that it is advantageous to acquire information by combining multiple ion sensors. The possibility of developing improved calibration models has been demonstrated using methods that combine PCR and PLS algorithms with neural networks [25]. Thus far, few attempts have been made to examine the potential of combining the properties of ANN with principal component analysis (PCA), that is, a principal component neural network PC-NN model (ANN model based on principle component scores) [46,47]. PC-NN, combining PCA with ANN, is another PCA-based calibration technique for nonlinear modeling between PCs and dependent variables [47,48]. The PC-NN analysis model was used for the estimation of rice nitrogen concentration from canopy hyperspectral reflectance [49]. Balabin et al. [50] reported comparison results between the linear model method and the nonlinear model method for predicting gasoline properties. When using multiple regression of spectral data, the PC-NN model not only reduces the data scale but also improves the results over the linear model.

The goal of this study is to develop a method that combines two types of sensor data using NIR spectroscopy and cobalt electrode reactions in order to quantitatively detect PO_4_^3−^ ion concentrations in a paprika nutrient solution. In addition, it focuses on developing a phosphate ion concentration prediction model using a combination of ANN, PLS and PCA data based on the possibilities indicated by preceding studies. NIR data were combined with PCR score data and raw EMF data from three cobalt electrodes to remove the multidimensional problem of the input spectrum data, where an artificial intelligence algorithm was employed. In the development of the calibration model, comparative verification was performed using PCR, PLS, ANN and PC-NN.

## 2. Materials and Methods

### 2.1. Preparation of Nutrient Solution Sample

In this experiment, materials were prepared with the goal of creating a nutrient solution for paprika (*Capsicum annuum* L.), which is a crop that can be cultivated using circulating hydroponics. The paprika nutrient solution was prepared according to the method (200 times concentration) presented by Sonneveld [51], as shown in Table 1. Here, the PO_4_^3−^ concentration was set such that changes in the ion concentration in the solution at an actual farm would be within the range of 10–1300 mg/L. To increase or decrease the PO_4_^3−^ concentration, (NH_4_)_3_PO_4_ was added or the amount of KH_2_PO_4_ was lowered. In total, 80 experimental samples were created, of which 56 were used for training and developing the calibration and verification models and 26 were used for testing. The actual concentration values of the created samples were analyzed at the National Instrumentation Center for Environmental Management (NICEM, Seoul National University, Seoul, Korea), a standard testing organization. Based on this, a standard for judging the accuracy of the prediction model was established. In the Appendix A (Table A1) is presented the specific concentrations of the samples determined by standard analyzer. NO_3_^−^, K^+^ and PO_4_^3−^ will hereafter be written without the charges, that is, NO_3_, K and PO_4_.

### 2.2. Data Acquisition and Analysis Using Cobalt Electrodes and NIR Spectroscopy

This study performed experiments for determining phosphate ion quantity combining spectrum data and EMF signal values from the cobalt electrodes in order to propose a sensor fusion system. The system follows the concept illustrated in Figure 1. First, PCR and PLSR from NIR data were used to design each calibration model. The principle score values obtained from these were combined with the cobalt electrode signals and used in the ANN to evaluate the performance of the model.

#### 2.2.1. Phosphate Prediction Model Using Cobalt Electrode

**Figure 2:**
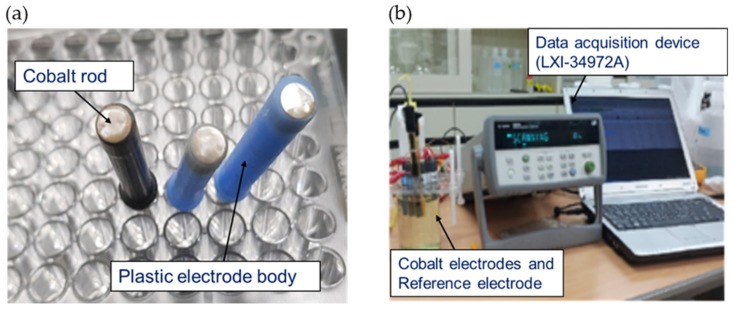
Developed cobalt rod-based electrodes (**a**) and the measurement system of cobalt electrodes used in this study (**b**).

In view of the results of a previous study [16], a cobalt metallic material was selected as a material that selectively reacts to phosphate in the dihydrogen phosphate (H_2_PO_4_^−^) ion form. The experiments employed a principle by which electrical potential occurs when the solid cobalt replaces dihydrogen phosphate in the solution. As shown in Figure 2a, the phosphate selective electrode was created from a 99.95% pure cobalt rod with a diameter of 5 mm (Sigma-Aldrich, St. Louis, MO, USA), by cutting it to a length of 6 mm and soldering it to a 1 mm copper wire. The cobalt connected to the copper wire was inserted into a PE plastic body with an external diameter of 6 mm and an internal diameter of 5 mm and silicon was injected between the cobalt metal and the plastic to prevent other materials from making contact. The surface of the cobalt electrode was polished using two types of sandpaper (400 and 1500 grit) and a soft cloth to obtain a stable reaction in the solution. The cobalt electrode with a polished surface was stabilized by soaking it in twice-distilled water for 20 min before use, until the EMF became uniform. Subsequently, a KHP 0.025M concentration buffer solution was mixed into the solution sample at a 5:1 ratio and the pH changes were fixed. Three electrodes and a standard electrode were used to obtain the EMF values of each sample. To achieve this, a multichannel data logger (LXI-34972A, Agilent, Santa Clara, CA, USA) was employed (Figure 2b).

#### 2.2.2. Prediction Model of Phosphate Ion by NIR Spectrum PLSR and PCR

A NIR spectrometer (model: NIR 128L, Control Development Inc., South Bend, NI, USA) and a halogen lamp (LS-1, Ocean Optics Inc., Largo, FL, USA) were used to measure the NIR band penetration spectrum for the prepared samples. The nutrient solution samples were placed in a 5 mL quartz cuvette to obtain the penetration spectra. Preprocessing was performed on the obtained 900–1700 nm NIR data in order to perform the PLSR and PCR analysis.

The moving average, standard normal variate (SNV) and multiplicative scatter correction (MSC) of the spectra were calculated using MATLAB R2016b (MathWorks, Natick, MA, USA). It has been reported that in the NIR band the PO_4_ absorption wavelength bands are 930–960 nm and 1020–1120 nm [52,53].

The cobalt electrodes and NIR spectra were used to develop a phosphate ion concentration prediction model through a PLSR and PCR 10-fold cross-validation method using MATLAB R2016b. The coefficient of determination (R^2^) and RMSE were used to confirm the accuracy of the model.

### 2.3. Development of Data Fusion Model for Phosphate Sensing

**Figure 3:**
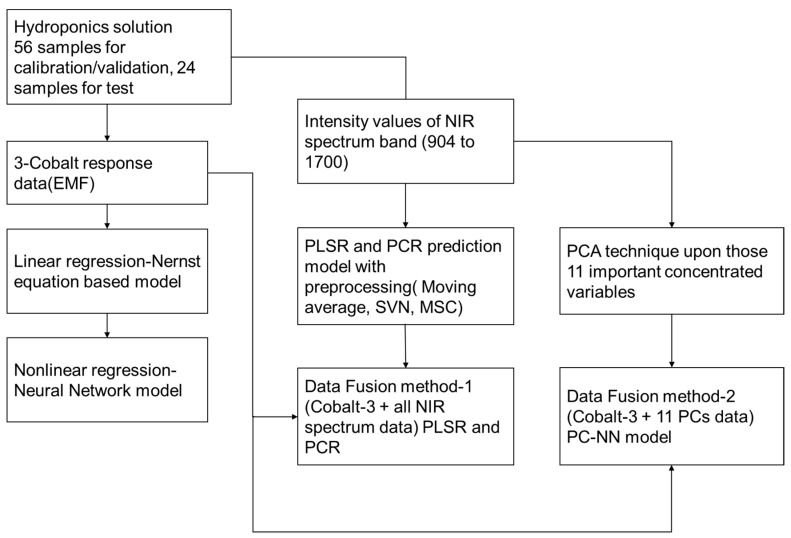
Block diagram of the proposed algorithms.

Figure 3 represents the method of developing the regression model for each phosphate ion through the cobalt electrode and NIR spectral data. Two fusion methods were used in this study, as indicated in the block diagram. First, PLSR regression analysis was performed by appending the data of three cobalt electrodes and all NIR band data to one array as input. This method is the same as the fusion method proposed by La et al. [38], with the only difference being the replacement of expected values by the reaction value of cobalt. The second is the PC-NN method.

**Figure 4:**
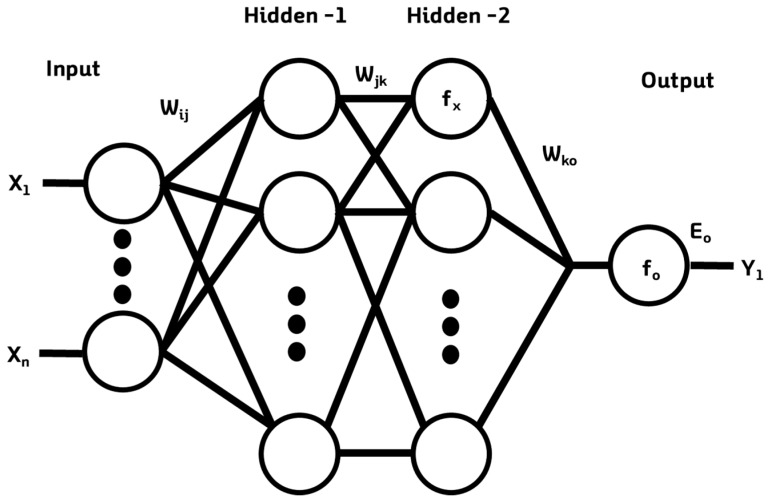
Example of a feed-forward back-propagation ANN model.

The architecture of all ANN and PC-NN systems presented in this paper consists of four layers of neurons or nodes, which are the input layer, two hidden layers and the output layer. ANNs are generally composed of input, hidden and output layers. The signals that enter the input layer are transmitted to the hidden layer and output layer through functions that transmit signals between the layers. Figure 4 illustrates the structure of an ANN with a simple multilayer structure [54]. In input layer I, the input variable xi connects to each single input node and each input node transmits a weight value W_ij_ to hidden layer J. Here, the sum of the W_ij_ values is denoted by a_ij_, with each node of the hidden layer as the standard. The weight values are replaced with values that have been changed through the activation function f(x) in each hidden layer node. In each node of the hidden layer, the W_ko_ weight value coefficient and the bias values are transmitted to the output layer O and transmission occurs for the value a_jk_. The node values of the output layer are determined by the function f_o_ and the error value Eo is obtained via the differences between these values and the label values (y) in the training data. The Levenberg–Marquardt algorithm, which is a gradient descent method for avoiding local minima and overfitting, was used for training.

This process is repeated for each sample. The error between a transmitted signal and the actual values for training is transmitted in the reverse direction to refresh the weight values for each layer through a generalized optimization method. The back-propagation algorithm was used for training the networks. We adopted various functions as the transfer function (activation) for all neural networks [55]. The activation functions used in this study are described in Table 2. The main network parameters (number of nodes in the hidden layer, learning rates and momentum) were subjected to various trials to find the best fitting condition, which are described in Table 3.

## 3. Results

### 3.1. Cobalt Electrode Reaction

**Figure 5:**
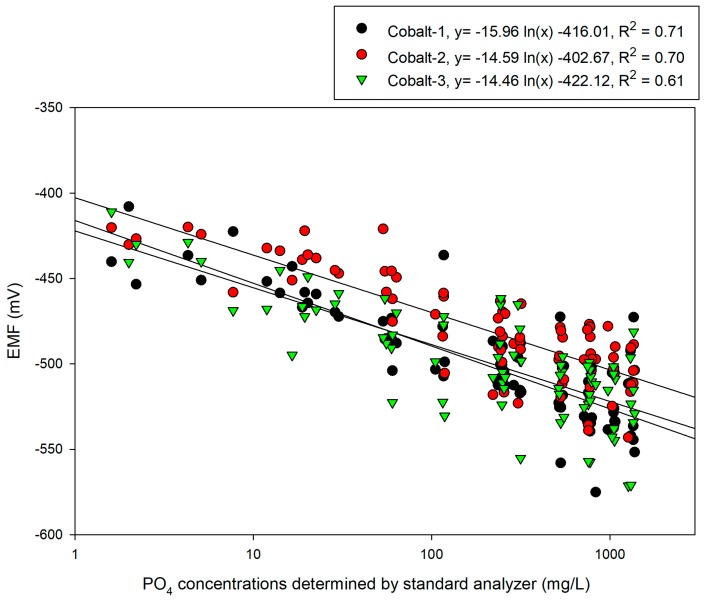
Calibration plot for linking a cobalt-electrode response to PO_4_^3−^ concentrations.

**Figure 6:**
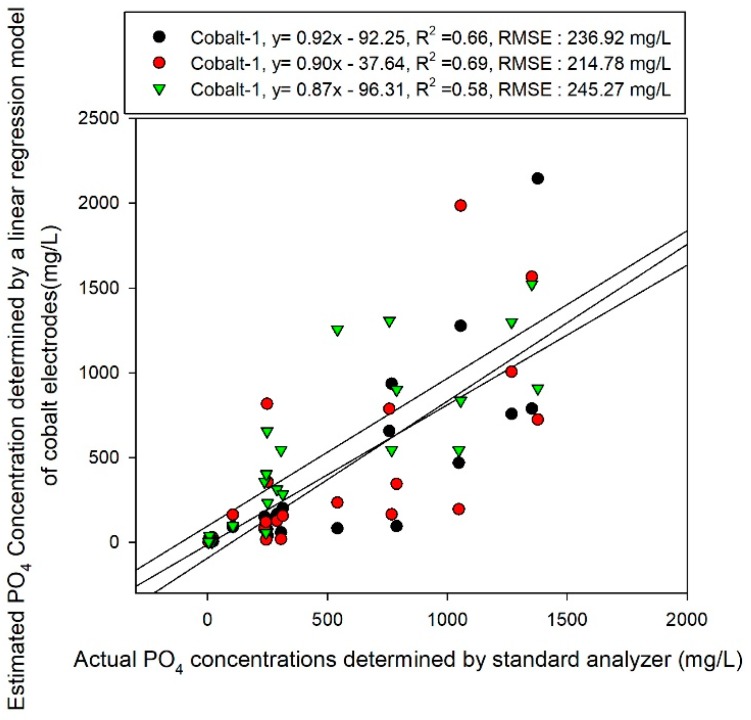
Test sample prediction results using the Nikolsky–Eisenman equation-based model with the electromotive force (EMF) response of cobalt electrodes.

As shown in Figure 5, three cobalt electrodes were used to detect the reactions of the electrodes for 56 obtained samples, assuming that the range of phosphate concentrations that can exist within the nutrient solution is 10–1300 mg/L. A calibration plot was created in the form of the Nikolsky–Eisenman equation, which shows the logarithmic relationship between the concentration and electrode EMF. The coefficient of determination was at a level of 0.61–0.71 and it was fairly difficult to operate the actual sensors. It was also observed that a fair amount of deviation in the reactions exists between electrodes and a normalization method is accordingly required. These problems can be attributed to changes in the states of the electrode conditioning. The cobalt electrodes were polished before the experiments and conditioning was performed. Subsequently, after around six hours, the experiments were completed. Around one minute was required for the sample measurement and five minutes were taken to clean the electrodes between samples. Figure 6 shows the results of predictions of cobalt EMF data for the concentration of test samples based on the calibration equation obtained in Figure 5. The coefficients of determination were obtained relatively poor (0.58–0.66) and the RMSEs obtained for each cobalt electrode ranged from 214.78 to 245.27 mg/L. There is a very strong possibility that the oxidation layer (CoO) that formed on the cobalt electrodes was altered during this time. To resolve this problem, it will be necessary to present automated measuring equipment as well as conditioning and a buffering solution that can maintain the oxidation layer over a long period.

**Figure 7:**
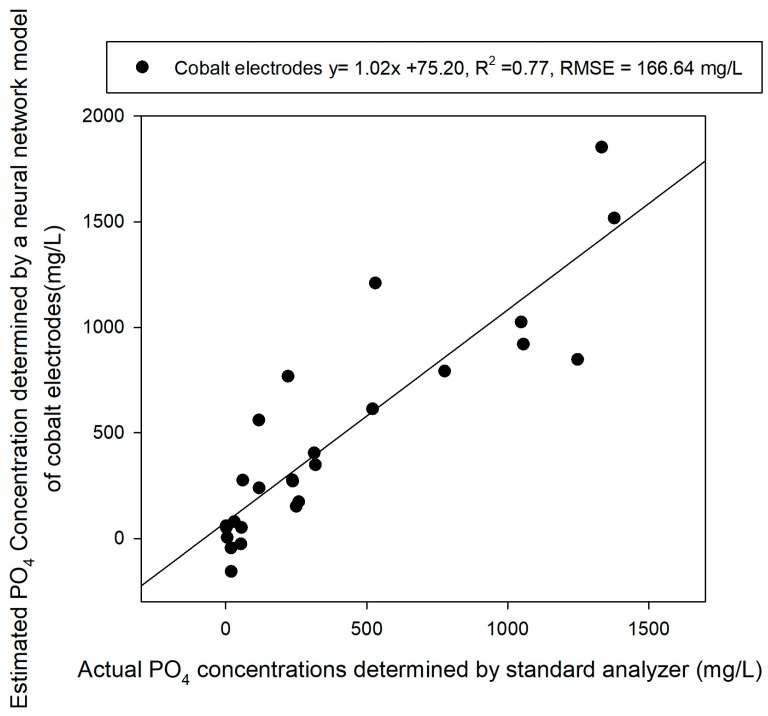
Test sample prediction results using ANN analysis with the EMF response of cobalt electrodes.

Figure 7 shows the results of the same test sample obtained through the prediction model developed through the ANN training model. The ANN model uses two hidden layers with 12 and 10 nodes and Tanh and the Logistic function were used. This optimal condition is obtained through trial and error. We obtained a RMSE of 166.64 mg/L in the test sample with a coefficient of determination of 0.77, which was slightly better than the results obtained using the linear calibration model (Figure 7).

### 3.2. Development of PLSR and PCR Prediction Model using NIR Spectroscopy Data

**Figure 8:**
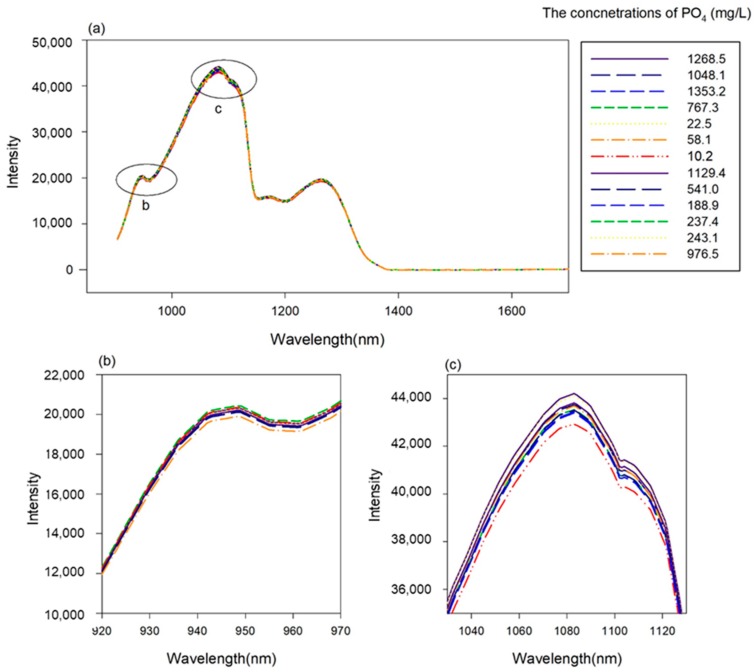
Intensity values of the NIR band for each PO_4_^-^ concentration. (**a**); PO_4_ absorption wavelength band in 920 to 970 nm (**b**); PO_4_ absorption wavelength band in 1040 to 1140 nm (**c**).

**Figure 9:**
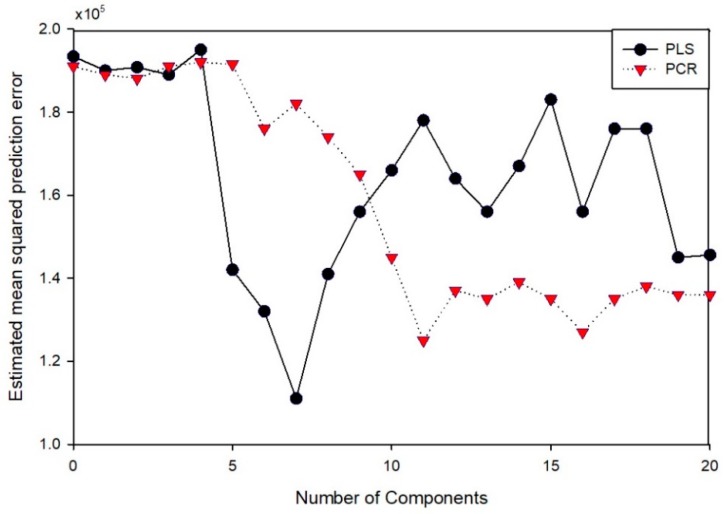
Comparison of partial least squares regression (PLSR) (Moving average 11and standard normal variate (SNV)) and principal components regression (PCR) (Moving average 11 and multiplicative scatter correction MSC) performance according to number of principal components.

Figure 8a shows the raw data from the NIR band of 904–1700 nm, which were obtained to detect phosphate quantities in the paprika nutrient solution specimens. Figure 8b and c show the 955 nm and 1100 nm portions, respectively, which are the PO_4_ absorption wavelength bands reported in previous studies [52,53]. The previously mentioned preprocessing method was applied to develop the calibration model using PLS and PCR. The results are presented in Table 4. In the case of PLS, when smoothing (moving average 11) and SNV were applied, the best results were obtained among the preprocessing methods, producing a coefficient of determination (R^2^) of 0.64 and a validation coefficient of determination (R^2^) of 0.34. However, these results did not represent a large improvement compared to the raw data and they are not satisfactory for sensors. The results of the PCR model were similar. The best results in PCR are the results of preprocessing using smoothing (moving average 11) and MSC. In these results, the detection method using the NIR band could not function as a sensor for detecting quantities of phosphate ions in the nutrient solution. However, the PCR results had a slightly higher coefficient of determination and lower RMSE than the PLSR results and thus the NIR data fusion was performed using the PC score data obtained via PCR (Figure 9).

### 3.3. Phosphate Detection via Data Fusion

As previously mentioned, this study attempted to develop prediction models through data fusion. First, the raw spectrum data and the cobalt EMF data were combined to develop machine learning models. Second, the PC-NN model was employed for the fusion method. In this method, PC components obtained in NIR spectrum data and EMF data from the cobalt electrodes were fused. Figure 9 presents a performance comparison based on the principal component distribution functions, where the best performance was obtained when 11 PCR score components were used. The fusion data were employed again in a model comparison using PLSR, PCR and PC-NN.

**Figure 10:**
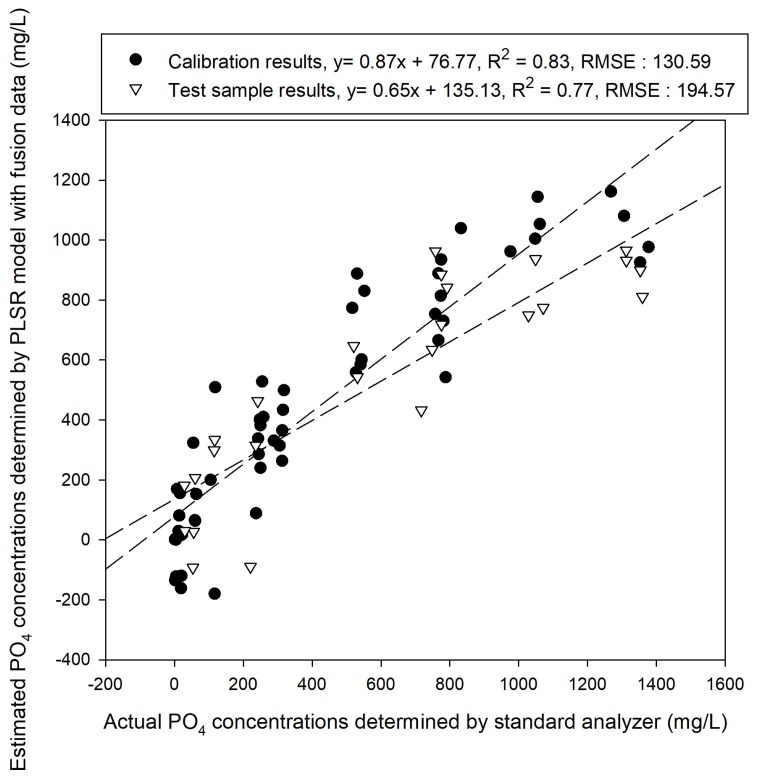
Calibration and test sample prediction results with PLSR analysis performed using the combined data.

**Figure 11:**
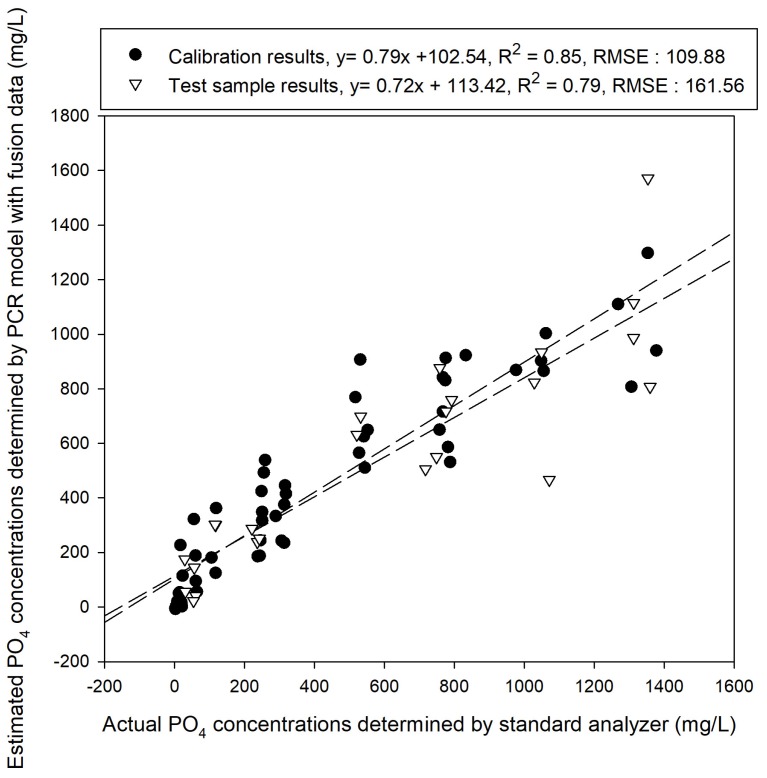
Calibration and test sample prediction results with PCR analysis performed using the combined data.

**Figure 12:**
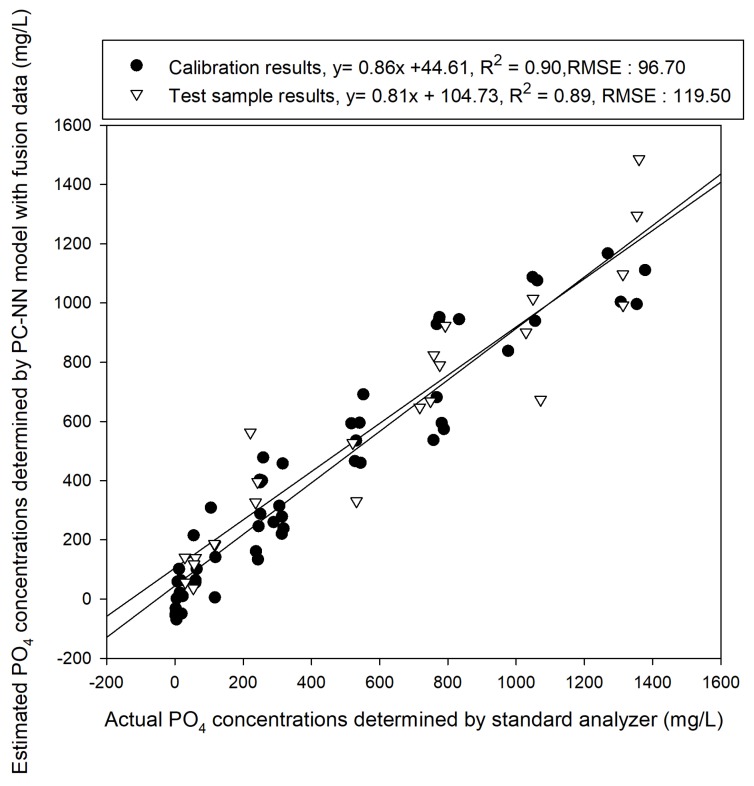
Prediction results of the test samples and calibration model developed using a PC-NN model on the combined data.

Figure 10, Figure 11 and Figure 12 illustrate the performances for predicting samples that were learned based on the developed models. As shown Figure 10, the calibration model developed using PLSR on the fused data had a slope of 0.87 and a coefficient of determination R^2^ of 0.83. The results predicted using the test samples had a slope of 0.65 and an offset of 135.13, with R^2^ of 0.77 and RMSE of 194.57 mg/L. When the fused data were analyzed via PCR, results of the same level were obtained. Figure 11 shows that the slope of the PCR calibration model was 0.79 and its R^2^ value was 0.85. The prediction results of the test samples using the PCR calibration model exhibited R^2^ of 0.79 and RMSE of 161.56 mg/L, showing that the performances of both PCR and PLSR were improved compared to those before addition of cobalt electrode EMF data. However, the accuracy was not as high as expected.

Table 5 shows both training and test results under each proposed training conditions for the model development through PC-NN. The best results were obtained using the Logistic and Tanh functions with 30 and 25 nodes and the learning rate was 0.001. As shown in Figure 12, the developed best fit model had a slope of 0.86 and an offset of 44.61, with a coefficient of determination of 0.90 and an RMSE of 96.70. The results of predicting the test samples using the developed model had a slope of 0.81 and an offset of 104.73, with a coefficient of determination (R^2^) of 0.89 and an RMSE of 119.50 mg/L.

## 4. Discussion

In this study, we applied two measurement methods for quantitative detection of phosphate ions and developed a more precise prediction model through the data fusion technique for each method.

In the first application, the results of the cobalt electrode exhibited low repeatability. The best regression model had R^2^ of 0.71, which is much lower than the results reported in previous studies [16,56]. There is a very high possibility that the oxidation layer (CoO) that formed on the cobalt electrodes was altered during this time. To resolve this problem, automated measuring equipment as well as conditioning and a buffering solution that can maintain the oxidation layer over a long period are required. Previous studies have linearly compensated a series of electrochemical response data through the two-point normalization method using two known concentration samples [4,57]. However, this method has a disadvantage in that two standard samples must be prepared at all times by fitting nonlinear data. The sample obtained in this manner has the potential of improving the results using the corresponding compensation method but we used raw data in this study.

As the second application target, transmission spectrum data of the NIR band was used. As shown in Figure 8, the reaction was confirmed at 960 to 970 nm and 1100 nm bands, which have been reported in previous research [31,32,33]. In addition, regression models were developed through preprocessing methods with the PLSR and PCR methods, which were mainly used in previous studies [22,25]. The best results were obtained using the PCR-based method with a coefficient of determination of about 0.64. Each electrochemical sensing and spectral response contributed to the improvement of the phosphate sensing performance. We expected the good sensitivity of the electrochemical sensing and the selective reacting of the spectral response to be ideally complementary and the improvement of fused two type of sensing methods s is confirmed in Figure 10 and 11. In Table 4, We compared the performance of the models developed under the seven cases of hidden node conditions. Although the statistical significance of hidden conditions was not confirmed, the results of PC-NN based models generally showed slightly better results than those of the conventional linear model (PLSR, PCR) As the phosphate ion concentration in the nutrient solution is a system with strong nonlinearity, PLSR or the linear compensation method was found to have a limitation but the nonlinear regression was possible in the artificial neural network structure. The amount of calculations increases as the number of nodes increases and thus learning requires a large amount of time for cases such as spectrum data, which have many input variables. To resolve this problem, a method has been proposed. This method applies a principal component analysis model to reduce the input dimension elements of ANNs and its effectiveness has been confirmed by several studies [34]. We obtained better results for nonlinear models than linear models in data fusion in Figure 12 The calibration results showed R^2^ of 0.90 and the results of validating the test samples showed R^2^ of 0.89. This means that, as reported in previous studies [33,41,50], complexity that cannot be defined as hypotheses in data fusion appear as learning results of nonlinear models. For example, model interferences due to drift signals can be eliminated through the interaction between electrodes.

The two applications we have used are the Cobalt-electrochemical method and the NIR spectrometer, which are easy to apply in the field as a system that can predict the phosphate concentrations after measuring within 1 min except for conditioning and preparation time. In terms of cost, the Cobalt-based electrode and NIR spectrometer are both cost-effective and sustainable platform. Furthermore, it is determined that it is possible to reduce the cost by optimizing the design on site and several effective multi-spectral band spectrometers.

The phosphate ion concentration in the paprika nutrient solution is generally within the range of 100–500 mg/L and Figure 10, Figure 11 and Figure 12 show that the range of used samples adequately allows the observation of abnormal changes in phosphate ions. In particular, the measurement samples targeted nutrient solutions applicable to hydroponic cultivation. Previous research [12,13,58] have proposed an ion sensing based nutrient control system, through which nutrient replenishment can be performed more precisely by adding this phosphate sensor to the control system. Furthermore, assuming that there are unexpected insufficiencies or excesses in the phosphate salts owing to technical problems with the pump or user errors when adding salts, the range in which the phosphate ions can vary in hydroponics should be considered to be 10–1200 mg/L and within this range, phosphate ions can be predicted via the proposed method. However, the prediction performance is somewhat insufficient in the low concentration band and this remains to be addressed. Furthermore, as previously mentioned, automated measuring equipment and hardware are required to effectively integrate the two measurement methods. (citation) If this equipment is employed to detect phosphate ion concentrations in nutrient solutions in real time, quicker and more accurate evaluation of phosphate ion absorption patterns of crops can be expected. In this manner, important information on crop growth can be obtained. This approach is applicable not only to nutrient solutions but also to measurement equipment for examining soil pollution caused by organic components.

## 5. Conclusions

This study used a data fusion method to develop PLSR, PCR and ANN models for predicting phosphate ion concentrations in a paprika nutrient solution. The main findings of the study can be summarized as follows.

A calibration plot was developed for measuring solution samples using three cobalt electrodes and the results showed a negative logarithmic correlation between the electrodes’ EMF and the concentration. However, a deviation existed between the three electrodes and there were limitations to their prediction performance, as indicated by their R^2^ values of 0.58, 0.6 and 0.69, respectively. Therefore, the effectiveness of their application to the prediction was very low.

NIR band penetration spectrum data were obtained from the paprika nutrient solution samples and used to develop prediction models via the PLSR and PCR methods. Smoothing, SNV and MSC spectrum preprocessing were applied but they did not achieve accurate prediction performance, with an R^2^ of 0.64 and a verification RMSE of 253 mg/L. As such, the effectiveness of their application was also low.

Eleven compressed results were extracted from the NIR spectral data based on the PCA score and they were fused with the cobalt electrode EMF data to create a new data set. PLSR, PCR and PC-NN were used on the data set to develop phosphate ion prediction models. The calibration sample results showed that the PLSR and PCR models achieved better performances (R^2^ of 0.83 and 0.85, respectively) than models without data fusion. The results of measuring the test samples for verification showed R^2^ values of 0.77 and 0.79. Nevertheless, the ANN prediction model achieved better results than the other two models. The calibration model showed an R^2^ of 0.90 and the test model showed an R^2^ value of 0.89 and RMSE of 119.5 mg/L. Here, no special compensation or normalization methods were used for the electrodes and the spectral data were not preprocessed.

From the results obtained by this study, the presented fusion method was found to facilitate effective detection of phosphate. Furthermore, rapid measurement of phosphate ions in a circulating hydroponic nutrient solution and effective management of the nutrient solution can be expected using the proposed method. In addition, a method for effectively fusing two different types of sensor via an artificial neural network is presented. This method is expected to be used in the future to develop more accurate sensors in a variety of fields.

## Figures and Tables

**Table 1:** Compositions of chemical compounds used for preparing 200 times concentrated solutions for hydroponic cultivation of paprika [24].

Tank	Chemicals Used	Amount (Per 2 L)
A	KNO_3_	48 g
	5[Ca(NO_3_)_2_ · 2H_2_O]NH_4_NO_3_	140.4 g
	NH_4_NO_3_	9.6 g
	EDTA-Fe(EDTAFeNa·3H_2_O)	1.29 g
B	KNO_3_	48 g
	KH_2_PO_4_	27.2 g
	Mg(NO_3_)_2_·6H_2_O	6.4 g
	MgSO_4_ · 7H_2_O	49.4 g
	H_3_BO_3_	0.31 g
	MnO4 · 5H_2_O	0.484 g
	ZnSO_4_ · 7H_2_O	0.228 g
	CuSO_4_ · 5H_2_O	0.04 g
	(NH_4_)6Mo_7_O_24_ · 4H_2_O	0.018 g

**Table 2:** Types of functions used in neural network hidden nodes.

Name	Equation		Derivative	
Logistic	$f\left(x\right)=\frac{1}{1+e^{-x}}$	(1)	$f^{\prime }\left(x\right)=f\left(x\right)\left(1-f\left(x\right)\right)$	(2)
Tanh	$f\left(x\right)=\frac{2}{1+e^{-2x}}-1$	(3)	$f^{\prime }\left(x\right)=\frac{1}{x^{2}+1}$	(4)
ReLu	$f\left(x\right)=\{\begin{array}{l} 0\;for\;x<0\\ x\;for\;x\geq 0 \end{array}$	(5)	$f^{\prime }\left(x\right)=\{\begin{array}{l} 0\;for\;x<0\\ 1\;for\;x\geq 0 \end{array}$	(6)

**Table 3:** Conditions of the hidden node for developing a phosphate ion prediction model through ANN.

Cases.	Hidden-1		Hidden-2		Learning Rate
	Number of Nodes	Activation Function	Number of Nodes	Activation Function	
1	30	Logistic	25	Tanh	0.002
2	35	Tanh	25	ReLu	0.001
3	30	Tanh	20	Logistic	0.003
4	35	Logistic	30	ReLu	0.001
5	35	ReLu	25	Logistic	0.002
6	30	Logistic	25	Tanh	0.001
7	25	ReLu	20	ReLu	0.003

**Table 4:** Comparison of PLS and PCR performance analysis results for various preprocessing methods applied to the NIR spectrum data.

Pre-Processing	PLSR		PCR	
	Calibration Results	Test Results	Calibration Results	Test Results
Raw	RMSE: 261.06	RMSE: 378.58	RMSE: 253.08	RMSE: 353.37
	R^2^: 0.64	R^2^: 0.28	R^2^: 0.64	R^2:^ 0.36
Smoothing moving average 11	RMSE: 261.41	RMSE: 370.58	RMSE: 327.45	RMSE: 394.71
	R^2^: 0.64	R^2^: 0.31	R^2^: 0.44	R^2^: 0.20
Smoothing moving average 11 + SNV	RMSE: 260.66	RMSE: 342.02	RMSE: 328.48	RMSE: 381.70
	R^2^: 0.64	R^2^: 0.34	R^2^: 0.44	R^2^: 0.26
Smoothing moving average 11 + MSC	RMSE: 282.05	RMSE: 370.35	RMSE: 260.30	RMSE: 329.06
	R^2^: 0.58	R^2^: 0.30	R^2^: 0.64	R^2^: 0.37

**Table 5:** Comparison of training and test results under each proposed training conditions for the model development through PC-NN.

Cases.	Training Results	Test Results
1	R^2^: 0.84, RMSE: 103.23	R^2^: 0.81, RMSE: 136.52
2	R^2^: 0.87, RMSE: 121.24	R^2^: 0.85, RMSE: 135.22
3	R^2^: 0.79, RMSE: 143.17	R^2^: 0.74, RMSE: 174.62
4	R^2^: 0.88, RMSE: 107.23	R^2^: 0.82, RMSE: 127.23
5	R^2^: 0.80, RMSE: 154.24	R^2^: 0.75, RMSE: 182.87
6	R^2^: 0.90, RMSE: 96.70	R^2^: 0.89, RMSE: 119.50
7	R^2^: 0.89, RMSE: 102.33	R^2^: 0.75, RMSE: 144.76

## Appendix A

**Table A1:** Phosphate ion concentrations of 80 samples analyzed via ion chromatography.

No.	Concentrations (mg/L)	No.	Concentrations (mg/L)	No.	Concentrations (mg/L)	No.	Concentrations (mg/L)
1	1268.5	21	1055.7	41	781.8	61	792.1
2	1048.1	22	243.1	42	552.0	62	116.8
3	1353.2	23	244.8	43	1.6	63	236.3
4	767.3	24	1377.7	44	2.2	64	1313.2
5	22.5	25	18.8	45	315.7	65	242.2
6	5.1	26	20.2	46	313	66	717.5
7	105.1	27	63.5	47	543.9	67	1071.6
8	19.4	28	2.0	48	318.0	68	1029
9	11.9	29	767.3	49	117.1	69	1049.4
10	7.7	30	60.3	50	254.9	70	1312.5
11	541	31	118.4	51	14.1	71	775.9
12	788.1	32	54.7	52	516.8	72	1353.7
13	288.9	33	1061.8	53	250	73	115.6
14	237.4	34	16.5	54	258.5	74	55.8
15	248.1	35	527.2	55	774.1	75	30.1
16	250.0	36	530.6	56	775.5	76	53.5
17	313.5	37	1306.5	57	1359.9	77	28.7
18	4.3	38	832.9	58	520.8	78	749.1
19	305.7	39	976.5	59	758.7	79	221.0
20	757.9	40	59.6	60	60.4	80	531.9
